# Contamination of UK firefighters personal protective equipment and workplaces

**DOI:** 10.1038/s41598-022-25741-x

**Published:** 2023-01-10

**Authors:** Taylor A. M. Wolffe, Anna Clinton, Andrew Robinson, Louis Turrell, Anna A. Stec

**Affiliations:** 1 Centre for Fire and Hazards Sciences, University of Central Lancashire, Preston, Lancashire PR1 2HE UK; 2 Royal Preston Hospital, Lancashire Teaching Hospitals NHS Foundation Trust, Preston, Lancashire PR2 9HT UK

**Keywords:** Environmental sciences, Risk factors, Cancer prevention

## Abstract

Firefighters’ personal protective equipment (PPE) is a potential source of chronic exposure to toxic contaminants commonly released from fires. These contaminants have also been found in fire stations. However, little research characterises the routes via which fire contaminants travel back to fire stations. The UK Firefighter Contamination Survey provides information on firefighters’ PPE provision, decontamination, and storage practices. All serving UK firefighters were eligible to take part in the survey, which comprised 64 questions. A total of 10,649 responses were included for analysis, accounting for roughly 24% of the UK’s firefighting workforce. Results revealed that most firefighters (84%) de-robe contaminated PPE/workwear after re-entering the appliance cab. There was a significant decreasing tendency to send PPE for cleaning after every incident with increasing seniority of role, length of service, and fire attendance frequency. Around one third of firefighters cleaned PPE after every incident. A number of issues were linked to external professional cleaning services, e.g. shrinkage, fit, turn-around time, and stock of reserve/pooled PPE. PPE storage was found to be a potential source of cross contamination, with almost half of firefighters (45%) indicating clean and dirty PPE is not stored separately. More than half of firefighters (57%) stored fire gloves (an item sent for professional decontamination by only 19% of firefighters, and never cleaned by 20%) within other items of PPE such as helmets, boots and tunic/trouser pockets. The survey’s results can be used to target gaps in decontamination measures within UK Fire and Rescue Services.

## Introduction

Firefighters are directly exposed to potentially large quantities of toxins on a regular basis when attending fires^1,2^. This puts firefighters at an increased risk of developing adverse health outcomes and emphasises the importance of managing those risks by implementing controls which protect against exposure^3,4^. Personal protective equipment (PPE) is one of the most important control measures for firefighters^5^ as it is often impossible to implement higher-level controls in the dynamic and transient working conditions presented by fire incidents^6^ and where firefighters are (necessarily) in close proximity to exposure sources^4^.

In the UK, a firefighters’ PPE ensemble typically comprises fire tunic (jacket) and trousers, boots, helmet, fire gloves, fire hood and respiratory protective equipment^7^ (and may vary according to hazard^6^). Additionally, a layer of “workwear” (t-shirt, trousers etc.) is worn beneath this ensemble. While PPE is designed to keep firefighters safe from several hazards (e.g. extreme heat, surface wetting etc.)^8^, a growing body of literature calls into question the efficacy of its protective function with regards to contaminant exposure^5,9^. For example, Mayer et al.^10^ demonstrated potential diffusion of toxic gases/vapours through firefighters’ turn-out jackets, reporting measured concentrations of benzene, toluene and naphthalene inside firefighters’ turn-out jackets which matched concentrations measured outside^10^. Studies have also documented particulates (including polycyclic aromatic hydrocarbons (PAHs)) penetrating interfaces of PPE ensembles, which may then transfer directly to the skin and/or workwear^11^.

Currently, there is no UK standard which requires firefighters’ PPE to protect against chemical or biological agents (i.e. particulates, fire gases etc.)^8^, remaining an area in need of further research and development^5^. In other countries, such research/development has led to innovations such as particle-blocking fire hoods, which have been adopted in the USA^12^. Still, there are other routes via which PPE may contribute to firefighters’ exposure to contaminants that require careful consideration. For example: when de-robing, handling, and storing contaminated PPE^13^; PPE that is gassing-off^14^; when donning or handling PPE which has been insufficiently decontaminated, or has been cross contaminated through storage or during domestic laundering cycles, etc^15^. If not adequately controlled, these exposures may negate any future improvements in the design and protective function of firefighters’ PPE ensembles.

Recent studies have also focused on measuring contaminant levels in the air/dust at fire stations where firefighters and support workers spend a considerable amount of their time^16,17^. Higher concentrations of polycyclic aromatic hydrocarbons, organophosphorus flame retardants, polybrominated diphenyl ethers etc. have all been found in the air and/or in dust collected from fire stations^16–18^, most likely brought back to the station on contaminated PPE or equipment after firefighting activities. Particular attention has recently been paid to per-fluorinated compounds in fire stations, as these compounds are not only used in firefighters' PPE, but are also a major ingredient of certain firefighting foams^19,20^.

While current research serves to evidence the extent of PPE and workplace contamination, relatively little research focuses on the routes via which contaminants are allowed to travel from fire incidents back to stations. The UK Firefighter Contamination Survey addresses this evidence gap by assessing firefighters’ experiences and behaviours on a range of topics including health (cancer^21^ and mental health^22^), PPE contamination/decontamination practices, and culture/habits and training^23^. This manuscript explores firefighters’ capacity for occupational exposure to fire contaminants through their PPE (i.e. PPE provision, maintenance, storage, fit etc.) and workplace. The results of the survey are intended to inform targeted contamination control measures within UK Fire and Rescue Services (FRSs), as part of a wider research project which aims to protect firefighters’ occupational health.

## Methods

### Survey design

The UK Firefighter Contamination Survey consisted of 64 questions (Supplementary File S1). Questions of specific relevance to this manuscript are outlined in Table S1 (demographics), Table S2 (PPE) and Table S3 (workplace contamination). Remaining questions are analysed in other manuscripts^21–23^.

Branching logic was used to route participants through the survey based on answers to previous questions. The survey was piloted with a small subset of firefighters, and questions rephrased for clarity according to feedback. Ethical approval for the survey was granted by the University of Central Lancashire Ethics Committee, and all analyses were conducted in accordance with relevant guidelines and regulations.

The survey ran through Jisc software^24^, for a period of 3 months between November 2019 and February 2020. A link to the survey was distributed to participants via email through the Fire Brigades Union (FBU). The survey took approximately 20 min to complete and was supported by UK Fire and Rescue Services (FRSs) with respect to allowing firefighters dedicated time within their workday in which to complete it.

### Participants

All currently serving (i.e. excluding retired) UK firefighters were eligible to participate, regardless of specific role or employment contract type. Firefighters were invited to anonymously complete the survey online. Informed consent was obtained from all participants.

### Analysis

Free text answers were manually coded for analysis according to the most commonly appearing themes.

Analyses conducted for this manuscript predominantly involved generating summary statistics for survey questions/responses. Where appropriate, survey questions were cross-tabulated in order to explore trends or correlations between variables. Statistical tests encompassed the following: chi-squared test (for differences between survey and general population demographics), *z* score test for difference in proportions, Kendall correlation, crude odds ratios, and chi-squared test for trend. These analyses were conducted in Excel (Office 365), or by using the statsmodels.api module for Python 3^25^.

P-values for *z* scores and chi-squared tests are quoted in-text below. For Kendall correlation tests, the correlation coefficient (tau-b) is presented alongside the p-value, giving an indication of the strength of correlation on a scale of 0–1.

## Results and analysis

The responses of 10,649 firefighters were included in analyses (Supplementary File S2), representing approximately 24% of the UK’s total firefighter workforce (Supplementary File S3).

### Demographics

The demographic characteristics of surveyed firefighters are displayed in Table 1. Participants tended to be white, male firefighters between the ages of 35–54, who work wholetime contracts and attend between 1–2 fires per week and 1–2 fires per month (Table 1). Surveyed firefighters were not significantly different to the English firefighter population (roughly 7% of which are female) with respect to sex (p > 0.05)^26^. However, participants were significantly different with respect to age, ethnicity and employment type (p < 0.05)—whereby the survey under-represents younger age categories, retained firefighters, and firefighters who belong to an ethnic minority.

**Table 1:** Participant Demographics.

		Proportion of surveyed firefighters/% (n = 10,649)
Sex	Male	87.1
	Female	6.4
	Other	0.0
Ethnicity	White	94.0
	Mixed race	1.3
	Black	0.5
	Asian	0.4
	Other	0.3
Age	< 24	2.6
	25–34	19.6
	35–44	34.3
	45–54	38.5
	55 +	4.0
Employment type*	Wholetime	73.9
	Retained	11.5
	Wholetime/retained	10.7
	Flexi-duty	3.1
	Other	0.4
Role	Firefighter	59.2
	Crew manager	18.0
	Watch manager	16.9
	Station manager	3.9
	Group manager	1.1
	Area manager	0.2
	Principal manager	0.1
	Other	0.2
Avg. no. fires attend	I do not attend fires	1.1
	< 1 per year	0.3
	1–2 per year	4.1
	1–2 per month	38.8
	1–2 per week	37.0
	> 3 per week	17.4
Years of service	0–9	23.5
	10–19	40.1
	20–29	31.6
	30–39	3.9
	40 +	0.4

The proportion of total surveyed firefighters belonging to each demographic category.

*Wholetime firefighters work full-time contracted hours/shift patterns.

Retained firefighters do not have contracted hours but are kept on a paid “retainer” contract, remaining on-call for emergencies.

Wholetime/retained firefighters represent wholetime firefighters who work a second retained contract outside of wholetime hours.

Flexi-duty firefighters work full-time contracted hours in a more flexible shift pattern.

All UK Fire and Rescue Service (FRS) locations, except for the Isle of Scilly and Jersey, were represented in the survey (Supplementary File S2, Fig. S1). As might be expected given that London and Scotland are the largest FRSs in the UK, participants from London (13%) and Scotland (13%) FRSs made up the largest proportions of all surveyed firefighters.

### PPE age

**Figure 1:**
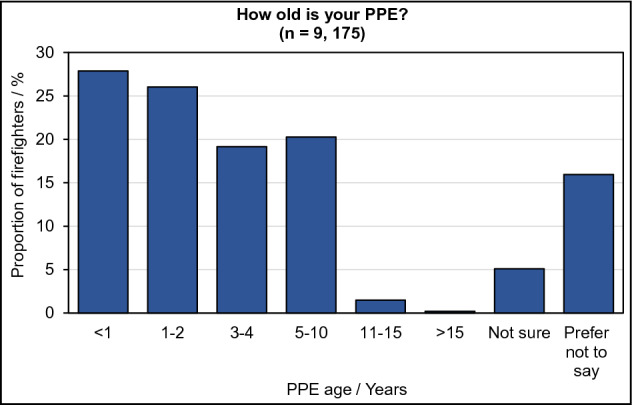
Age of UK Firefighters’ PPE. Proportion of firefighters with individually issued PPE indicating the age of their PPE.

Only firefighters with individually issued PPE were able to answer questions about the age of their PPE—the results of which are displayed in Fig. 1. The majority (86%) of surveyed firefighters had individually issued PPE. Approximately 13% of firefighters had PPE from a shared, “pooled stock”.

There was an increasing trend in PPE age with increasing years of service (tau-b = 0.13, p < 0.05), fire attendance frequency (tau-b = 0.10, p < 0.05), and seniority of role (tau-b = 0.05, p < 0.05). However, while the relationship between these variables is significant, the effect of one upon the other is very small.

### PPE fit

Almost 10% of all surveyed firefighters expressed concern over the fit of their PPE. Around 70% of these firefighters (n = 739) detailed their concerns as free text (Supplementary File S2, Fig. S2). The most common issues mentioned were:

- Poor PPE fit after washing; PPE shrinking after being sent for professional cleaning.
- Inconsistent sizing; fit varies between same item/size of PPE—e.g. due to different suppliers, varied PPE ages etc.
- Availability of different PPE sizes; poorly tailored to individual body dimensions, firefighters falling “between sizes”, poorly fitted to female bodies.
- Availability of spare or shared PPE; lack of stock necessitates wearing of ill-fitting PPE from pooled/reserve stock, e.g. while individually issued PPE sent away for professional cleaning.
- PPE irregularly (re-)fitted; fit not updated to match changing body shape/size of wearer.
- Uncomfortable or impractical; fit uncomfortable, restrictive, or makes wearer too hot.

Several items of PPE received specific mention amongst firefighters’ concerns over PPE fit:

- Gloves; generally ill-fitting and impractical.
- Boots; too tight, impractical.
- Helmets; generally ill-fitting or uncomfortable.
- Tunics; too short, small or tight.
- Leggings; too short, small or tight.
- Trousers; too short, small or tight.
- Braces/straps; too loose, requiring constant adjustment.

Firefighters using pooled PPE were significantly more likely to report concerns over fit compared to firefighters with individually issued PPE (crude OR = 2.5, 2.1–2.9). A significantly larger proportion of female firefighters expressed concerns over PPE fit compared to male firefighters (p < 0.05), being over 3 times more likely to report ill-fitting PPE than their male colleagues (crude OR = 3.1, 2.6–3.8).

### At the fire incident

**Figure 2:**
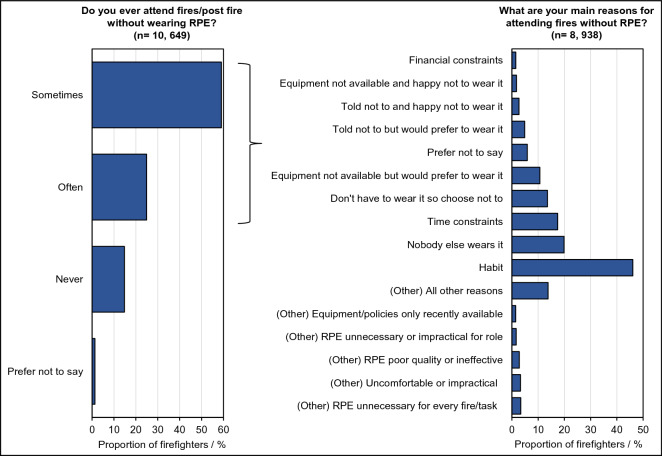
Attending fires without respiratory protective equipment. Proportion of total surveyed firefighters who attend fires without respiratory protective equipment (RPE), and their reasons for doing so. Note that firefighters were able to indicate more than one reason.

Firefighters were asked about their use of respiratory protective equipment (RPE) while attending fires, including at the early stages of a fire, and post-fire, Fig. 2.

Around 84% (n = 8938) of surveyed firefighters indicated that they often or sometimes attend fires without RPE (Fig. 2). The most common reasons were: habit (46%, n = 4122), “nobody else wears it” (20%, n = 1777) and “time constraints” (17%, n = 1559, Fig. 2).

Around 14% (n = 1245) of firefighters provided other reasons for not wearing RPE; most commonly indicating that RPE was not considered necessary for every task or fire incident. RPE was also described as “uncomfortable” and “impractical”, causing over-exertion, over-heating, difficulty breathing, difficulty communicating and/or hindering vision and movement. Several items of RPE (e.g. disposable dust masks) were also specifically cited as being poor quality, or unfit for purpose. Breathing apparatus (BA) was also cited as too heavy, impractical or time consuming for use at all incidents/post-fire activities.

### Returning from the fire incident

**Figure 3:**
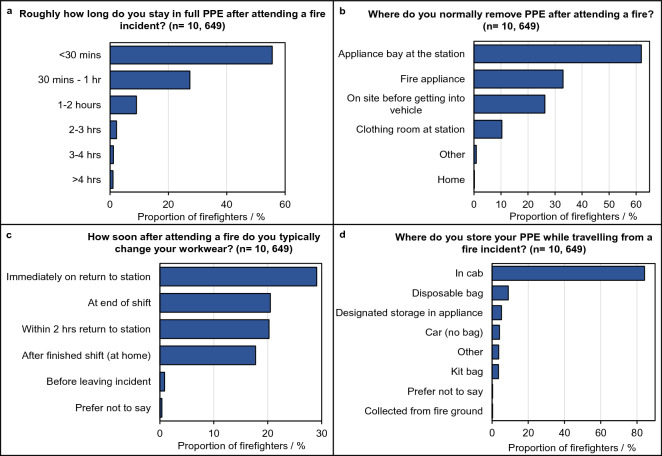
PPE de-robing and storage after fire incidents. Proportion of total surveyed firefighters indicating: (**A**) how long they would normally remain in PPE after attending a fire, (**B**) where they would normally de-robe PPE after a fire, (**C**) how long they would normally remain in workwear after a fire, and (**D**) where PPE is normally stored while travelling back from a fire incident. Note, firefighters were able to select multiple answers to the questions presented in this figure.

The proportion of firefighters remaining in PPE and workwear for specific lengths of time after attending a fire are displayed in Fig. 3 and Table S7 (Supplementary File S2).

In general, the proportions of firefighters remaining in PPE for given lengths of time did not significantly differ from expected across length of service, role and employment type categories (Supplementary File S2, Table S7). Most firefighters remained in PPE for up to an hour after attending a fire (Fig. 3A).

There was a slight increasing tendency for firefighters to remain in PPE for the shortest time periods (i.e. < 30 min) the more frequently they attend fires (Table S7). Firefighters attending fires on at least a weekly basis were significantly more likely to remain in PPE for < 30 min, while those attending fires on a monthly or yearly basis were significantly less likely to remain in PPE for < 30 min (Table S7).

Only 26% of surveyed firefighters would de-robe contaminated PPE on-site, before re-entering the appliance/vehicle cab. Most surveyed firefighters (62%) indicated that they would de-robe PPE in the appliance bay back at the station (Fig. 3B). There was a significant association (p < 0.05) between de-robing on-site and length of service, role, and fire attendance frequency—whereby senior and longer serving firefighters were increasingly likely to de-robe onsite, whereas firefighters attending fires more frequently were less likely to do so.

Less than 1% of total surveyed firefighters indicated that they would change their workwear before leaving the fire incident (Fig. 3C). Compared to the total surveyed population, firefighters with senior roles were significantly more likely to remain in workwear until the end of a shift or until they had returned home (Supplementary File S2, Table S8). Flexi-duty and retained firefighters were also more likely to remain in workwear for the longest time periods, while a significantly larger proportion of wholetime firefighters changed workwear soon after returning to the station (Table S8). Firefighters attending fires more frequently were also significantly more likely to remain in workwear until the end of a shift (Table S8).

The majority of surveyed firefighters (84%) indicated that PPE was stored in the cab of the appliance while travelling back from fires (Fig. 3D). Only 5% of firefighters indicated that there was a dedicated area of the appliance specifically for the storage of contaminated PPE. The main reason given for selecting “other” was that PPE was still worn while travelling back from an incident and was thus not “stored”.

### At the station

#### PPE decontamination

**Figure 4:**
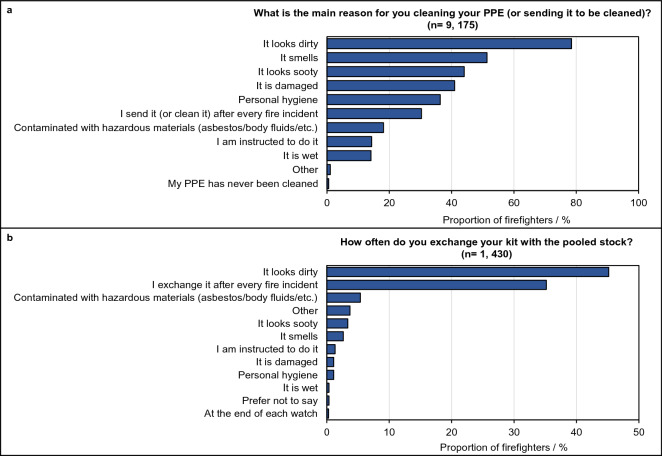
Reasons for cleaning/exchanging PPE in the UK Fire and Rescue Service. (**A**) The proportion of firefighters with individually issued PPE indicating their reasons for cleaning their PPE. (**B**) The proportion of firefighters with pooled PPE indicating their reasons for exchanging PPE with the pooled stock.

Figure 4A,B presents the most common reasons firefighters clean/exchange their PPE.

There was a decreasing tendency to clean PPE after every fire with increasing seniority of role, length of service or fire attendance frequency. The association between these variables was found to be significant (p < 0.05) in both firefighters with individually issued and pooled PPE (Supplementary File S2, Figs. S3,S4).

Most firefighters exchanging pooled PPE indicated that PPE was generally received clean and odourless (46%), or at least stained but otherwise clean and odourless (39%). However, 12% of firefighters indicated that pooled PPE either smells of smoke (5%) or appears dirty (7%).

#### PPE decontamination methods

Table 2 presents the proportion of firefighters who use specific PPE decontamination methods, and the proportion of firefighters who send PPE for professional cleaning on a regular basis.

**Table 2:** PPE cleaning methods and frequency of professional PPE cleaning in the UK Fire and Rescue Service.

	Proportion of firefighters / %			
	Tunic/Trousers (n = 9, 175)	Fire hood (n = 9, 175)	Gloves (n = 10, 605)	BA set (n = 10, 605)
**PPE cleaning method**				
I send it to be professionally cleaned	90	63	19	1
Washing machine	11	35	14	n/a
Brush	5	0	5	22
Hand wash with detergent/soap	2	2	43	80
Hand wash with water only	2	0	12	11
It is cleaned by a designated person on site	1	1	0	1
Other	1	0	1	5
Paper towel	1	0	2	35
These items do not get cleaned	0	3	20	4
Prefer not to say	0	0	0	1
**Professional cleaning frequency**				
More than once per week	0	0	0	n/a
Every week	1	1	0	
Every other week	4	3	1	
Every month	18	11	3	
Every other month	38	26	6	
Twice per year	26	23	8	
Once per year	6	8	4	
PPE not sent for professional cleaning	5	27	71	
Prefer not to say	2	2	1	

Firefighters were able to select multiple cleaning methods. Note that only firefighters with individually issued PPE were able to answer questions about the cleaning of tunic/trousers and fire hoods. Fire gloves are issued on an individual basis as standard, thus trends concerning fire gloves represent firefighters with individually issued and pooled stock PPE. It is not (currently) standard practice to send breathing apparatus (BA) for external professional cleaning in the UK.

The associations between demographic variables and the proportions of firefighters sending specific items of PPE for professional cleaning are presented in Supplementary File S2 (Table S9, and Figs. S5,S6). In general, the proportion of firefighters sending PPE for professional cleaning on at least a monthly basis decreased with length of service and seniority of role, but increased with fire attendance frequency for all items of PPE assessed.

Approximately 14% of firefighters with individually issued PPE indicated taking PPE home for cleaning (versus 85% who do not). Of those (n = 1, 314), 92% transported PPE home in a personal vehicle (versus 5% using a service vehicle and 2% using “other” modes of transport). Most (88%) confirmed using a home washing machine to launder their PPE (vs 11% who did not).

No significant associations were found between the tendency to take PPE home and length of service (p > 0.05), or seniority of role (p > 0.05). However, a significant association was found for fire attendance frequency (p < 0.05), whereby firefighters were increasingly likely to take PPE home for cleaning the more incidents they attend.

### Workplace contamination

Fire appliances were specifically mentioned by over 20% of firefighters providing free-text concerns over workplace/PPE cleaning (n = 4072), the interiors of which were cited as being poorly or rarely cleaned.

Around 85% of surveyed firefighters indicated that they work in stations with a designated zoning system for keeping clean/dirty areas separate, while 14% indicated no such system was in place. However, less than half of firefighters working in stations with a designated zone system (45%) thought that the designated area system was well adhered to.

The majority of surveyed firefighters also indicated that their workplace smelt of fire smoke (87%). Most firefighters (69%) indicated that this smell was only present immediately after a fire, however 18% of firefighters indicated that it was always present. Just 13% of firefighters indicated that their workplace did not smell of smoke.

#### PPE storage

**Figure 5:**
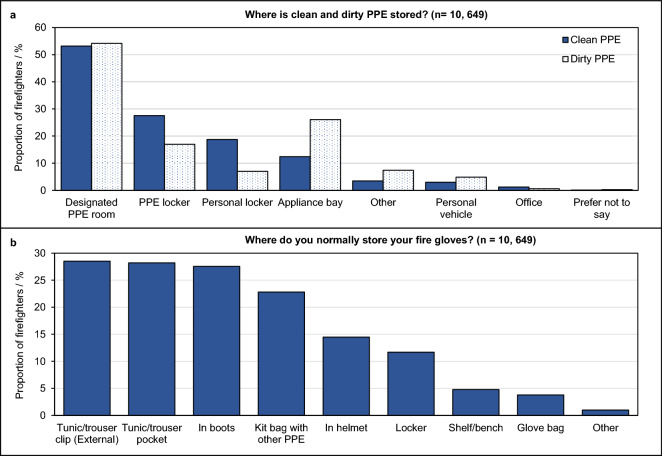
PPE storage in the UK Fire and Rescue Service. The proportion of total surveyed firefighters indicating: (**A**) where clean and/or dirty PPE is normally stored, and (**B**) where fire gloves are normally stored. Note that firefighters were able to select more than one location for the storage of clean/dirty PPE and fire gloves.

While 54% of surveyed firefighters indicated that clean and dirty items of PPE are stored separately, a similar proportion (45%) indicated that they are not. Storage locations for clean and/or dirty PPE are displayed in Fig. 5A. Firefighters with pooled PPE were significantly more likely to store PPE in appliance bays (clean or dirty) compared to firefighters with individually issued PPE (p < 0.05) (Supplementary File S2, Tables S10,S11), whereas a significantly larger proportion of firefighters with individually issued PPE stored PPE (clean or dirty) in personal vehicles (p < 0.05). Clean, individually issued PPE was also significantly more likely to be stored in a personal locker than clean, pooled-PPE (p < 0.05) (Tables S9,S10).

Notably, a significantly larger proportion of flexi-duty firefighters indicated storing dirty PPE in personal vehicles (p < 0.05) (65% versus around 5% for other employment types).

Storage locations for fire gloves are displayed in Fig. 5B. More than half of all firefighters (57%) store their fire gloves within other PPE items i.e. within helmets (14%), boots (28%) and/or tunic/trouser pockets (28%). Half of these firefighters indicated that clean/dirty PPE is not *s*tored separately (50%), and 21% indicated that their fire gloves were never cleaned at all. Similarly, 48% of firefighters storing fire gloves in a kit bag with other items of PPE indicated that clean/dirty PPE is not stored separately, with 20% indicating their gloves were never cleaned.

The practice of storing fire gloves in other items of PPE was significantly associated with length of service (p < 0.05), and fire attendance frequency (p < 0.05) (Fig. S7), whereby an increasing proportion of those serving longer and those attending fires frequently store fire gloves in other items of PPE. A significant association was also found for seniority of role, where a decreasing proportion of those in more senior roles stored their gloves in other items of PPE (Fig. S7).

## Discussion

### PPE provision and fit

The UK Firefighter Contamination Survey uncovered considerable variation in terms of PPE provision and decontamination practices. For example, analysis of free text suggested the standards and types of RPE issued to firefighters were highly variable, and that policies regarding the use of RPE were not enforced, leaving firefighters to make their own judgements. These judgements often deemed RPE unnecessary for open/outdoor fires, fire inspections/investigations, and post-fire activities e.g. damping down or tuning over. In fact, a majority of firefighters indicated that they would often/sometimes attend fires without any RPE- a risk of direct exposure to fire toxins.

The issuing of pooled versus individual PPE was another source of variation, with no clear trends in terms of demographics or geographic region for the use of pooled over individually issued PPE, suggesting variable PPE issuing policies in the UK Fire and Rescue Service.

The survey revealed concerns over the fit of pooled PPE, where firefighters were significantly (twice) more likely to report concerns over fit compared to firefighters using individually issued PPE. Similarly, poor fit and overall condition of reserved stock were given as reasons for firefighters avoiding sending personally issued PPE for professional cleaning on a more frequent basis (e.g. “We only have 2 sets and if we don’t have them we have to wear pooled gear which is ill fitting, old and not to the same standard.”).

It should also be noted that, regardless of whether PPE was pooled or individually issued, female firefighters were over 3 times more likely to express concerns over the fit of their PPE compared to male firefighters (OR = 3.1, 2.6–3.8). This issue has been repeatedly reported in literature concerning the ergonomics of firefighters’ PPE over the last two decades^27^, and appears to have persisted in the UK FRS despite the increasing proportion of female firefighters in the Service^26^.

As well as compromising comfort and mobility^27,28^, it is plausible that poorly fitting PPE may increase a firefighter’s risk of direct contaminant exposure e.g. as over-sized garments allow contaminants to penetrate gaps in interfaces, or under-sized garments leave skin/workwear exposed.

### PPE maintenance and decontamination

A larger proportion of firefighters (46%) wore PPE less than 2 years old. This is in line with the recommendations of the current British Standard (BS8617) which states that PPE should be replaced at least every 10 years (or sooner if damaged)^29^. However, around 2% of firefighters were found to have PPE which was over 10 years old, suggesting that this standard may not always be strictly adhered to. PPE has been found to become increasingly susceptible to damage as its age and number of wears increases. This puts firefighters at increased risk of exposure to fire toxins able to infiltrate damaged PPE layers. It is therefore vital that PPE is regularly inspected for damage.

The lighter colour of modern firefighting ensembles is designed to facilitate such inspection^30^—providing contrast against the darker colour of carbonaceous deposits and/or damaged areas. As such, “it looks dirty” was the most common reason surveyed firefighters (79%) gave for cleaning their PPE. However, toxic contaminants and gases which have permeated the fabric of PPE will not be visible. It is therefore important that PPE is thoroughly decontaminated after every fire incident, and not only when it visually appears dirty. Around one third of surveyed firefighters with individual/pooled PPE indicated that PPE is cleaned after every fire incident. Poor uptake of decontamination of such regularity has been documented in US firefighters, who raised concerns over PPE drying time and time-consuming decontamination procedures^31^.

Most firefighters indicated that PPE was sent for professional cleaning less than every month (Fig. 5). Further, not all items of PPE were routinely sent for professional cleaning. While the majority of firefighters sent tunic/trousers for professional cleaning (90% with individually issued PPE), fire hoods and fire gloves were sent less frequently by 63% and 19% of firefighters respectively.

Several barriers for sending PPE for professional cleaning were mentioned by firefighters as free text. The slow turn-around time of the professional cleaning service, in combination with a lack of PPE stock, was raised as a concern for firefighters who would otherwise be left without PPE while their sets were sent away. Firefighters also mentioned that PPE items would sometimes go missing after laundering, or that the wrong items were returned to them. However, the concern raised most often was that PPE tended to shrink after it was professionally cleaned, compromising fit e.g. “shrinks a great deal after cleaning so very often too small and have to get pool stock while waiting for suitably sized PPE”.

These barriers may offer explanation for the significant association between sending/cleaning PPE after every incident and fire attendance frequency, whereby firefighters were less likely to send/clean PPE after every incident the more frequently they attend fires. These firefighters may not have enough time to decontaminate their PPE between incidents and cannot afford to be without PPE (or left with ill-fitting PPE) for extended periods.

Firefighters in more senior roles and those who had served the longest were also increasingly less likely to clean/send their PPE after every incident. These significant associations may plausibly be explained by senior firefighters attending incidents in supervisory (rather than active firefighting) roles, where PPE does not become visibly contaminated and is therefore not cleaned. Long-serving, or incident-active firefighters may also have become desensitized to the threat of contamination or may not have received up-to-date training concerning the latest scientific evidence regarding the health effects of contaminant exposure^23^.

### PPE storage and cross contamination

The survey uncovered several potential routes via which contaminants can travel back from fire incidents to firefighters’ workplaces and their homes. Many of these routes are a result of cross contamination, and begin at the fire incident.

Most surveyed firefighters (84%) indicated that they would de-robe PPE worn during an incident after they had re-entered the appliance cab. This means that contaminants remaining on firefighters’ PPE can be transferred to the surfaces of the appliance cab, and then to fire stations, offices etc. (or personal vehicles and homes). Only 5% of surveyed firefighters indicated that there was dedicated storage within appliances for dirty PPE, or that dirty PPE was bagged in disposable plastic bags during storage (reported by 9% of firefighters). Firefighters’ free-text comments also revealed appliance interiors were rarely cleaned, allowing contaminants to build-up.

Significant proportions of firefighters (see Supplementary File S2, Tables S7,S8) indicated that they would remain in their PPE, or at least in their workwear, for an extended length of time after attending a fire incident, increasing opportunities for cross contamination.

Also of concern were the 14% of firefighters who indicated taking their PPE home to clean, storing contaminated PPE in a personal vehicle. This represents a direct route for contaminants to travel back to firefighters’ homes and their families. Flexi-duty firefighters were significantly more likely than expected to indicate storing soiled PPE in personal vehicles, most likely due to the nature of their employment. Special attention to the PPE storage needs of these firefighters is required to ensure that contaminated PPE is appropriately contained.

Laundering PPE in domestic-style washing machines has been found to be a key source of cross contamination, where contaminants may be transferred to laundry water and/or components of washing machines and later to other items of laundry^15^. This is concerning given the prevalence of firefighters using washing machines for laundering fire hoods (35% of firefighters) and gloves (14% of firefighters) in the UK FRS. These practices should be reviewed in order to minimise cross contamination risks.

One of the strongest cross-contamination examples uncovered by the survey, was the storage of fire gloves. Around 57% of firefighters indicated that they would store their fire gloves in other items of PPE, potentially allowing contaminants to transfer to the inside of helmets, boots and tunic/trouser pockets, which may all make direct contact with the skin of the wearer. What’s more, fire gloves are rarely sent for professional cleaning (19% surveyed firefighters).

Those who attended fires on more frequent bases, and who have served longer in the UK Fire and Rescue Service were increasingly likely to store fire gloves in other items of PPE. This is concerning given the greater number of direct exposure events these demographics have received, and indicates a lack of awareness and/or complacency over the comparatively subtle issue of cross contamination.

Less than half of firefighters (45%) indicated that clean and dirty PPE is not stored separately. Even if the clean and dirty PPE was stored separately, it was often kept in in the same room. Around 22% of firefighters indicated that there was no (or a poorly adhered to) system for separating clean/dirty areas within the station. In fact, a small proportion of firefighters (1%) indicated that PPE was stored in “clean” areas e.g. offices. This implies that, unless there are e.g. two PPE rooms, one dedicated for clean PPE and one for dirty PPE, the level of separation between clean and dirty PPE may not be sufficient to protect against cross contamination. For example, contaminants off-gassing from dirty PPE may diffuse to the area of the room in which clean PPE is stored. Similarly, removal of contaminants from the firefighter’s workplace surfaces may be less challenging if the level of separation between clean/dirty PPE is greater (e.g. storing dirty PPE outside of the station).

## Conclusions

The UK Firefighter Contamination Survey uncovered considerable variation in terms of PPE provision, cleaning and storage in UK Fire and Rescue Services. Several practices which increase the risk of direct contaminant exposure and/or cross-contamination were found to be prevalent amongst surveyed firefighters. As such, the survey identified several opportunities for immediate intervention:

- Policies concerning the use of RPE should be revised and better enforced. Firefighters should wear RPE at all fires, including during pre- and post-fire tasks. Different levels of protection may be required depending on the task/fire, thus adequate RPE should be available at all times.
- Firefighters in managerial roles should set an example to more junior colleagues in terms of adhering to PPE/workplace maintenance and decontamination practices.
- To prevent the passage of contaminants from fire incidents back to stations/homes, PPE and workwear should be de-robed on-site of the incident, before firefighters re-enter the appliance cab.
- Contaminated PPE/workwear should be:
  o Bagged in disposable plastic bags on-site before being placed in any (fire and rescue service) vehicle in order to contain off-gassing/surface contaminants and then stored in a designated area of the appliance/vehicle, or, preferably collected by professional cleaning services on-site.
  o All personnel who may have been contaminated at the incident, whether involved in firefighting activity or not, should undergo a process of personal decontamination before changing into clean workwear, such that only ever clean PPE/workwear makes contact with the interior of the appliance/vehicle cab.
- PPE storage and cleaning policies should prevent firefighters from cleaning PPE at home and/or transporting contaminated PPE in personal vehicles.
- Review of current policy for the decontamination of vehicles shows existing policy to be inadequate. FRSs should review their policy and develop suitable and sufficient decontamination procedures.
- The decontamination of appliance/vehicle interiors should be carried out in accordance with those improved procedures and should be conducted after every fire incident (however small) or other incidents where contamination is suspected.
- Storage of clean/ bagged dirty PPE also requires proper separation in order to prevent cross contamination, e.g. through enforcing clean/dirty zones in the station.
- Where PPE cannot or has not been collected on-site at an incident, dirty and bagged PPE should be sent for professional decontamination as soon as possible after every incident to ensure thorough removal of contaminants. This requires re-evaluating the policies and provisions concerning supplies of pooled PPE stock, PPE fit, and the length of the professional cleaning service.
- Regular training/ message-reinforcement on the health effects of contaminant exposure and/or best decontamination practices may raise firefighters’ awareness of cross contamination and serve to reduce the prevalence of practices such as storing fire gloves within other items of PPE, laundering fire hoods in home washing machines etc.
- Finally, standardisation is required in order to ensure effective decontamination practices. Promoting high-quality, nationally consistent policy on minimising exposures to fire toxins will help to reduce the incidence of occupational diseases and cancers in UK firefighters.

## Limitations

The UK Firefighter Contamination Survey was able to amass a sizeable sample of UK firefighters, potentially lending greater precision to the trends analysed in its results. However, the survey was unable to ask firefighters about all aspects of contaminant exposure or decontamination in specific detail. Thus, further trends in terms of PPE and workplace contamination may yet to be uncovered.

Additionally, the survey was cross-sectional in design; only able to assess firefighters’ current practices and exposures. It does not consider whether this current practice/exposure is representative of past practices/exposures. Thus, the survey cannot accurately account for how decontamination practices have changed over time and/or the reasons for this change.

The survey is potentially subject to participation bias, whereby firefighters with grievances against the Fire and Rescue Service may have been more likely to participate. Conversely, the survey may also under-represent firefighters’ personal experiences/adherence to decontamination practices as firefighters may plausibly have felt pressured to answer more “favourably” (for example indicating that they adhere to practices or station policies more frequently than they do in reality).

## Supplementary Information

Supplementary Information 1.

Supplementary Information 2.

Supplementary Information 3.

## Data Availability

The datasets generated and/or analysed during the current study are available from the corresponding author on reasonable request.
