# Supplementary material for: Contamination of UK firefighters personal protective equipment and workplaces
Source: Sci Rep. 2023 Jan 10;13:65. doi: 10.1038/s41598-022-25741-x (PMC9832125; doi:10.1038/s41598-022-25741-x)
Supplement: Supplementary file 2 — Supplementary Information 2. [file 41598_2022_25741_MOESM2_ESM.docx]

**Supplemental File S2**

Contamination of UK Firefighters Personal Protective Equipment and Workplaces

Taylor A. M. Wolffe^1^, Anna Clinton^1^, Andrew Robinson^1,2^, Louis Turrell^1,2^, Anna A. Stec^1,*^

^1^Centre for Fire and Hazards Science, University of Central Lancashire, Preston, PR1 2HE, UK

^2^Royal Preston Hospital, Lancashire Teaching Hospitals NHS Foundation Trust, Preston, Lancashire, PR2 9HT, UK

^*^Corresponding author: [aastec@uclan.ac.uk](mailto:aastec@uclan.ac.uk)

**Survey Participants**

All currently serving UK firefighters were eligible to take part in the survey. Therefore, the first question of the survey, *“Are you currently working as a firefighter in the UK?”*  was used to include/exclude survey responses from analysis. A total of 6 respondents indicated that they were **not** currently working as firefighters in the UK and were thus excluded from further analysis. A further 4 respondents identified themselves as retired in the free text answers they provided to survey questions and were thus excluded. Four hundred and seventy-one respondents left this initial question blank. Analyses were repeated with these respondents excluded. However, this did not alter the significance, direction or magnitude of trends identified in the manuscript. Thus, due to the nature of recruitment to the survey (i.e. via email to FBU members), these respondents were assumed to be currently serving UK firefighters and included in subsequent analyses. This left a total of 10,649 included respondents. This figure represents approximately 24% of the UK’s total Firefighter workforce (Supplemental File S3).

## **Geographic Distribution of Survey Responses**

**Figure S1: Geographic Distribution of Survey Responses and Response Rate.**FRSs are organised by participation rate (i.e. the proportion of each FRS’s firefighter headcount that took part in the survey) in descending order. The proportion of total surveyed firefighters each FRS represents is also presented.

**PPE fit**

**
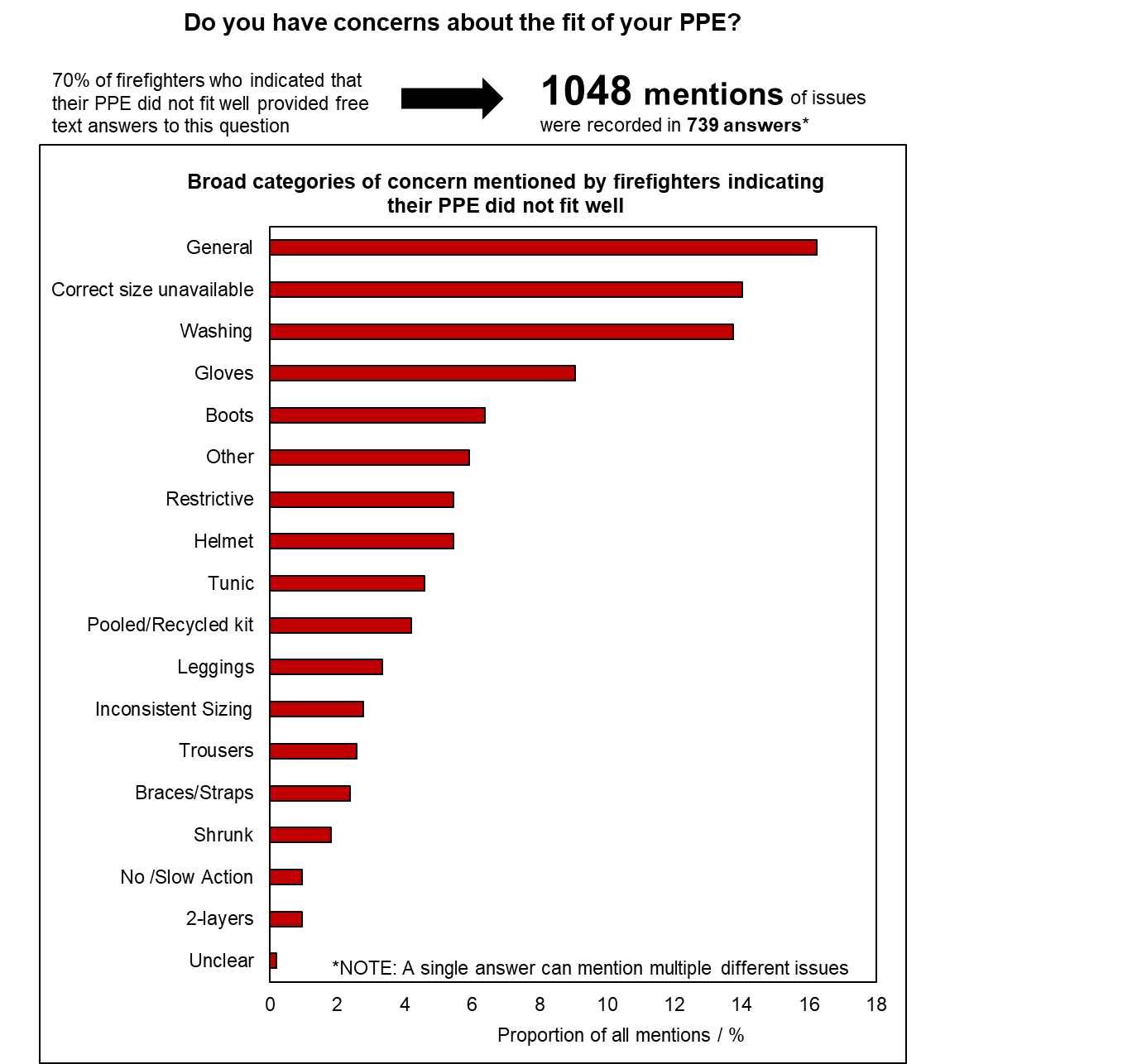
**

**Figure S2:** Concerns raised by firefighters leaving free text comments on the fit of their PPE.

**Table S7: Proportion of firefighters who remain in PPE for specific length of time (%).** Z-score test for difference in proportions is used to compare proportions of firefighters in specific demographic categories to the total (i.e. the overall proportion of all surveyed firefighters). Values in bold indicate statistical significance (p<0.05).

**Table S8: Proportion of firefighters who remain in workwear for specific length of time (%).** Z-score test for difference in proportions is used to compare proportions of firefighters in specific demographic categories to the total (i.e. the overall proportion of all surveyed firefighters). Values in bold indicate statistical significance (p<0.05).

**Returning from fire incidents**

**
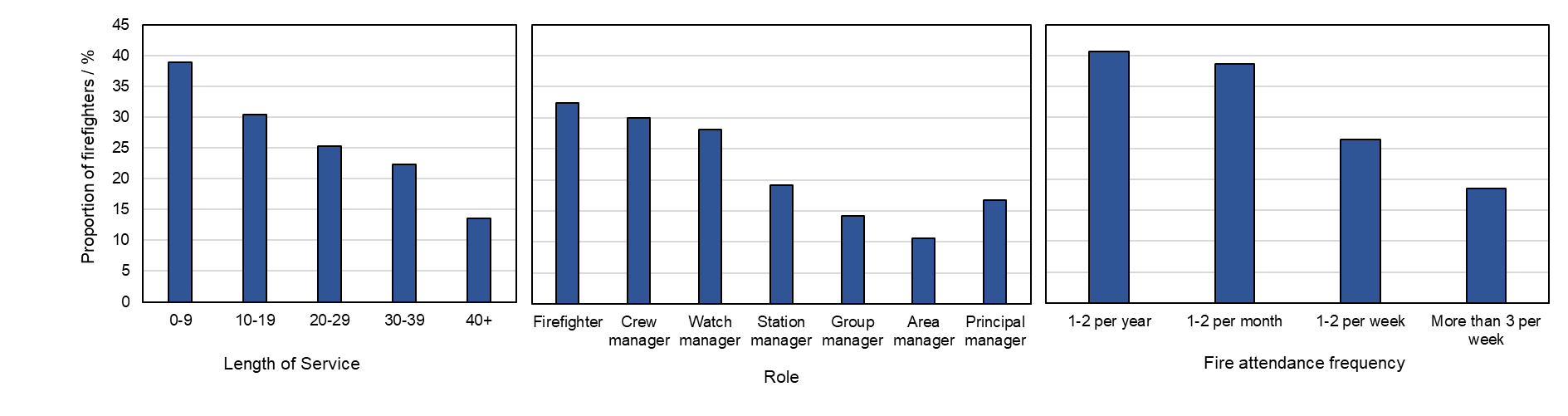
**

**Figure S3: Proportion of firefighters with individually issued PPE in each demographic category who clean their PPE after every fire incident.**

**
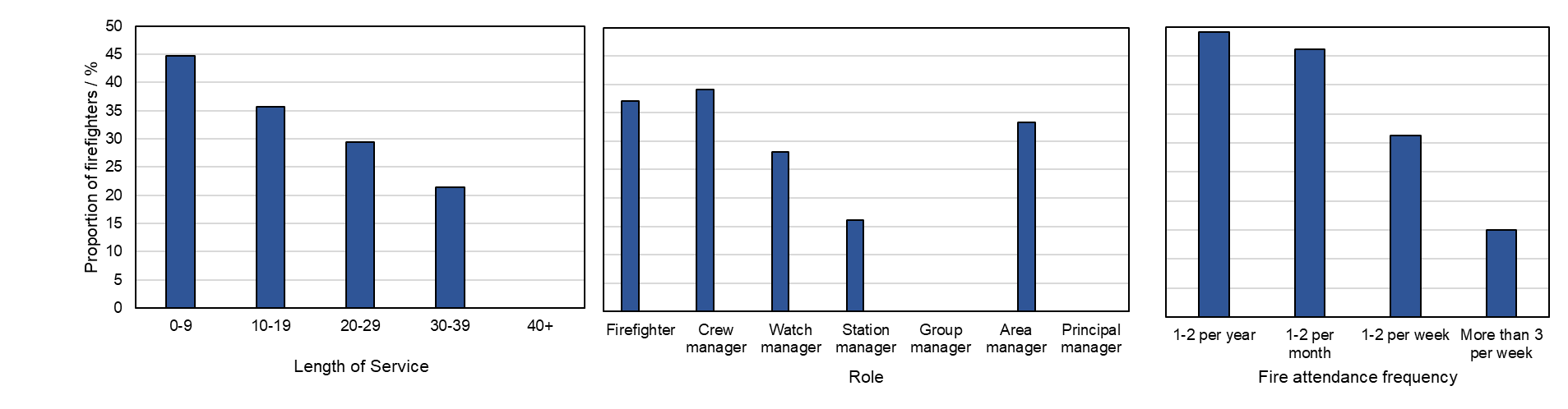
**

**Figure S4: Proportion of firefighters with pooled PPE in each demographic category who clean their PPE after every fire incident.**

|  |  | | Length of Service | | | | Seniority of Role | | | | Fire attendance frequency | | | |
| --- | --- | --- | --- | --- | --- | --- | --- | --- | --- | --- | --- | --- | --- | --- |
|  |  | | Tunic/trousers | Fire hood | Gloves | BA | Tunic/trousers | fire hood | Gloves | BA | Tunic/trousers | Fire hood | Gloves | BA |
| PPE sent for professional cleaning on at least a monthly basis | | χ^2^ | **2150** | **1321** | **381** | n/a | **1128.5** | **706.5** | **223.5** | n/a | **5947** | **3856** | **1108** | n/a |
|  |  | p | **<0.05** | **<0.05** | **<0.05** |  | **<0.05** | **<0.05** | **<0.05** |  | **<0.05** | **<0.05** | **<0.05** |  |
| PPE never cleaned | | χ^2^ | 29 | 353 | **2367** | **363** | 16 | **291.5** | 1510 | **190.5** | 59 | 732 | **5629** | 995 |
|  |  | p | >0.05 | >0.05 | **<0.05** | **<0.05** | >0.05 | **<0.05** | >0.05 | **<0.05** | >0.05 | >0.05 | **<0.05** | >0.05 |

**Table S9**: **Association between demographic variables and regularly sending PPE for professional cleaning, or never cleaning PPE at all.**
Results of chi-squared tests for trend are presented, df=1. Note that “PPE never cleaned” is a binary representation of whether firefighters selected “this item does not get cleaned” when asked about the methods used for cleaning PPE.


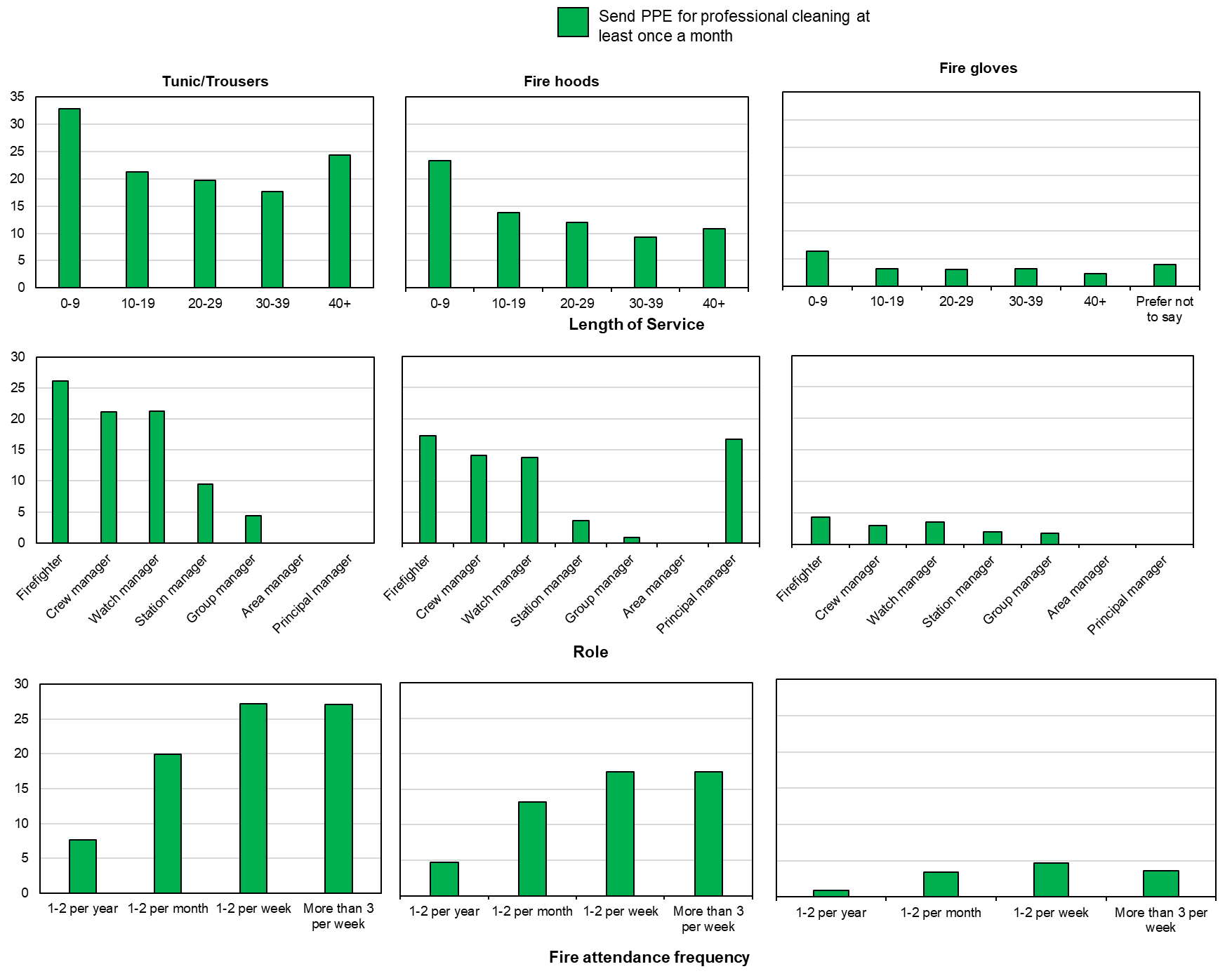


**Figure S5**: Proportion of firefighters in demographic categories who send PPE for professional cleaning more, or less frequently than once per month.

**
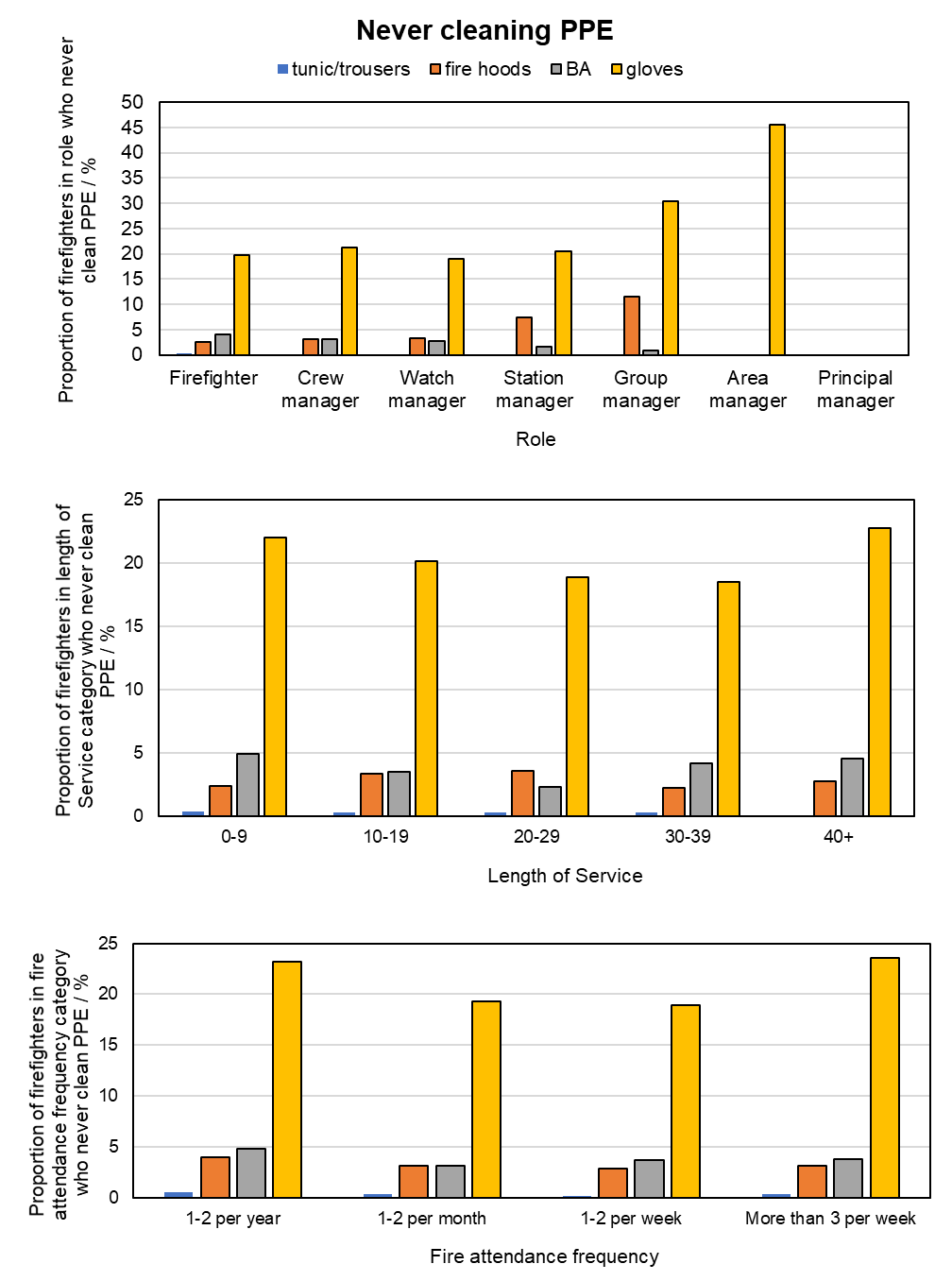
**

**Figure S6:** Proportion of firefighters in demographic categories who never clean their PPE.

| **Clean PPE Storage** | | |
| --- | --- | --- |
| Location | % Individually Issued PPE | % Pooled PPE |
| Appliance bay | **12.0** | **15.5** |
| Designated PPE room | 53.4 | 52.7 |
| Office | 1.3 | 1.0 |
| Other | 3.5 | 3.3 |
| PPE locker | **25.8** | **39.5** |
| Personal locker | **20.5** | **7.3** |
| Personal vehicle | **3.1** | **2.0** |
| Prefer not to say | 0.1 | 0.0 |

**Table S11:** **Proportion of firefighters who store clean PPE in specific locations (%).** Z-score test for difference in proportions is used to compare proportions of firefighters with individually issued vs. pooled PPE. Values in bold indicate statistically significant differences (p<0.05).

| **Dirty PPE Storage** | | |
| --- | --- | --- |
| Location | % Individually Issued PPE | % Pooled PPE |
| Appliance bay | **25.1** | **32.5** |
| Designated PPE room | 54.0 | 56.2 |
| Office | 0.5 | 0.9 |
| Other | 7.3 | 8.3 |
| PPE locker | **17.3** | **15.2** |
| Personal locker | 7.2 | 6.0 |
| Personal vehicle | **5.0** | **3.5** |
| Prefer not to say | 0.2 | 0.1 |
|  |  |  |

**Table S11:** **Proportion of firefighters who store dirty PPE in specific locations (%).** Z-score test for difference in proportions is used to compare proportions of firefighters with individually issued vs. pooled PPE. Values in bold indicate statistically significant differences (p<0.05).

**
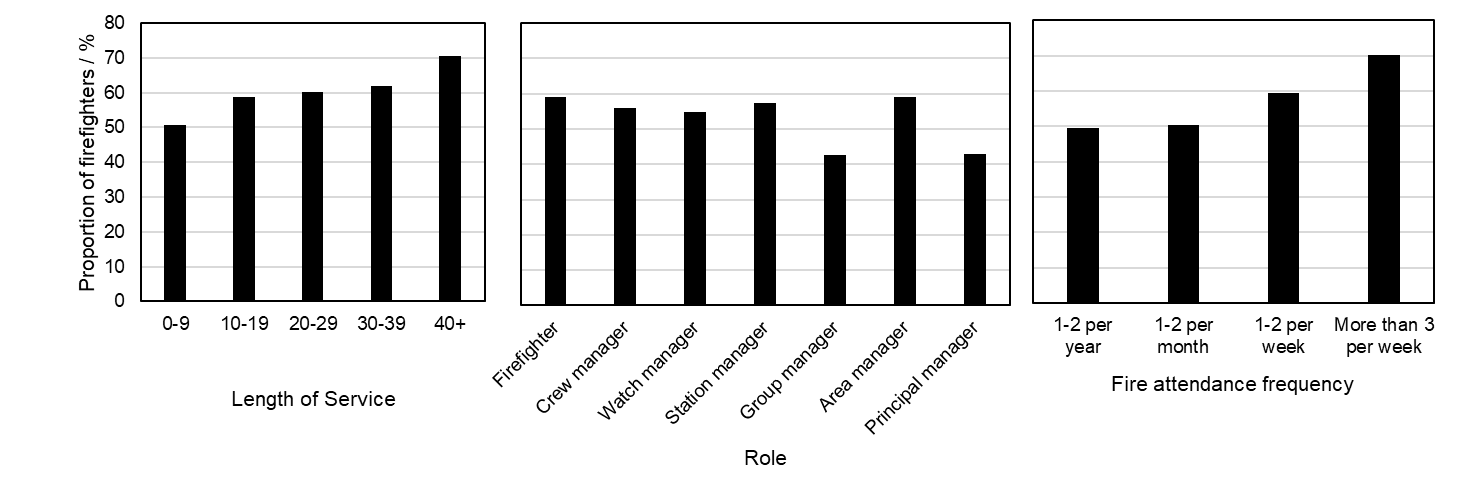
**

**Figure S7: Proportion of firefighters in each demographic category who store their fire gloves in other items of PPE.**
